# The effect of game-based in comparison to conventional circuit exercise on functions, motivation level, self-efficacy and quality of life among stroke survivors

**DOI:** 10.1097/MD.0000000000028580

**Published:** 2022-01-14

**Authors:** Mohd Naqiuddin Johar, Nor Azlin Mohd Nordin, Aznida Firzah Abdul Aziz

**Affiliations:** a Physiotherapy Program, Center for Rehabilitation and Special Needs Studies, Faculty of Health Sciences, Universiti Kebangsaan Malaysia, Kuala Lumpur, Malaysia; b Physiotherapy Unit, Hospital Putrajaya, Putrajaya, Malaysia; c Department of Family Medicine, Faculty of Medicine, Universiti Kebangsaan Malaysia, Cheras, Kuala Lumpur, Malaysia.

**Keywords:** function, motivation level, quality of life, self-efficacy, stroke

## Abstract

**Introduction:**

Stroke survivors are commonly at risk of functional decline, which increase their dependency in activities of daily living and eventually affects their motivation level, self-efficacy, and quality of life. Circuit exercise has been shown to be useful in enhancing functional performance and quality of life of chronic stroke survivors. There is a need to review the existing “usual circuit exercise” and develop a better approach, such as game-based circuit exercise. Training in enriched and fun environment may possibly further promote neuroplasticity. However, evidence on inducing fun element in the existing circuit exercise among stroke survivors is limited. Also, no studies are available to date which report the benefit of circuit exercise on stroke survivors’ self-efficacy and motivation level. Therefore, this study aims to assess the effectiveness of game-based circuit exercise in comparison to conventional circuit exercise on functional outcome (lower limb strength, postural stability and aerobic endurance), motivation level, self-efficacy and quality of life among stroke survivors. This study also aims to assess whether the outcomes gained from the 2 interventions could be sustained at week 12 and 24 post-trial.

**Methods:**

This is an assessor-blinded randomized control trial comparing 2 types of intervention which are game-based circuit exercise (experimental group) and conventional circuit exercise (control group). Based on sample size calculation using GPower, a total number of 82 participants will be recruited and allocated into either the experimental or the control group. Participants in the experimental group will receive a set of structured game-based exercise therapy which has the components of resistance, dynamic balance and aerobic exercises. While participants in the control group will receive a conventional circuit exercise as usually conducted by physiotherapists consisting of 6 exercise stations; cycling, repeated sit to stand, upper limb exercise, lower limb exercise, stepping up/down and walking over obstacles. Both groups will perform the given interventions for 2 times per week for 12 weeks under the supervision of 2 physiotherapists. Outcomes of the interventions will be measured using 30-second chair rise test (for lower limb strength), Dynamic Gait Index (for postural stability), 6-minute walk test (aerobic capacity), Intrinsic Motivation Inventory questionnaire (for motivation level), stroke self-efficacy questionnaire (for self-efficacy) and Short Form-36 quality of life questionnaire (for quality of life). All data will be analyzed using descriptive and inferential statistics.

**Discussion:**

This study will provide the information regarding the effectiveness of including game elements into circuit exercise training. Findings from this study will enable physiotherapists to design more innovative exercise therapy sessions to promote neuroplasticity and enhance functionality and quality of life among stroke survivors under their care.

**Trial registration:**

Australian New Zealand Clinical Trials Registry, ACTRN 12621001489886 (last updated 1/11/2021)

## 1 Introduction

Stroke is a significant health problem and cause of disability among adults worldwide. It was reported that, in 2019 there were nearly 101 million prevalent cases of stroke and 143 million disability-adjusted life-years (DALYs) due to stroke globally.^[1]^ Generally, mortality rate of acute stroke has declined with the advancement of medical care; most countries now witness an increasing number of stroke survivors hence those living with disability.^[2]^ This increased prevalence of stroke-related disability which requires continuous and long-term rehabilitation and healthcare support places great burden on healthcare system in most countries especially in the developing region.^[3]^

Rehabilitation remains the mainstay of poststroke management, aiming to minimize impairment and disability, and improve functional independence among the stroke survivors. Physiotherapy, being a main part of the multidisciplinary rehabilitation program plays a vital role through the poststroke stages. Physiotherapeutic interventions for poststroke patients include therapeutic exercises, electrophysical agents, manual therapy, virtual reality therapy, mirror therapy, robotic therapy and special techniques such as motor relearning program and Bobath.^[4–7]^ The service is provided during hospitalization for acute and sub-acute stroke, and normally continued in out-patient settings once the stroke patient is discharged home.

Therapeutic exercise with a focus on task specific training is the most utilized physiotherapy modality for stroke survivors.^[8]^ It can be delivered either through individual or group session; with the group session normally conducted in a form of circuit class exercise. During circuit exercise session, a group of three or more participants is supervised by 2 to 3 physiotherapists in performing repetitive practice of a number of tasks which arranged in several workstations in sight of each other.^[4,9]^ Circuit exercise for poststroke patients is normally provided 2 to 3 times per week and continued for as long as 1 month or more postonset depending on the individual patient's needs.^[10–16]^

Circuit exercise has been found to improve stroke survivors’ functions and quality of life.^[10–16]^ However, the conventional form is seen as less enriched and lacking in fun. Training in enriched and fun environment can promote neuroplasticity, in addition to facilitate personalized motivation and cease stress and anxiety among stroke survivors.^[17–20]^ Nonetheless, no studies have incorporate design-for-fun games environment into the existing circuit exercise. Past studies about circuit exercise have also not included motivation and self-efficacy, 2 important determinants for recovery among stroke survivors^[21]^ in their assessment of intervention outcome despite the fact that stroke survivors frequently demonstrate decline in these component and affect their functional independence over time.^[21–26]^ There is a need to review the existing conventional circuit exercise and offer a more enriched environment by adding multisensory stimuli and cueing, limb integration and cognitive stimulation to further increased neuroplasticity potentials such as game-based circuit exercise.^[27]^ Therefore, the purpose of this study is to determine the effectiveness of game-based circuit exercise in comparison to conventional circuit exercise on functional outcome, motivation level, self-efficacy and quality of life among stroke survivors. This study also aims to assess whether the outcomes gained from the 2 interventions could be sustained at week 12 and week 24 post trial.

## 2 Methods

### 2.1 Study design and setting

**Figure 1:**
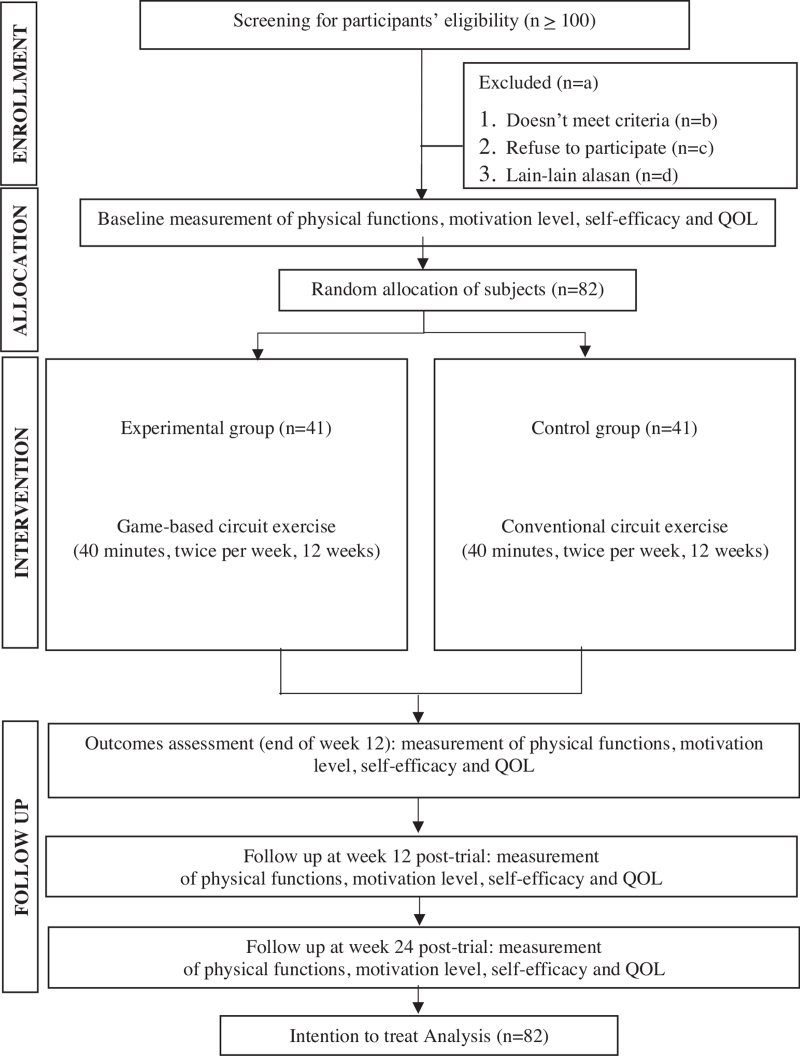
Consort diagram of the study.

This is an assessor-blinded randomized control trial which compare 2 types of intervention, namely game-based circuit exercise (experimental group) and conventional circuit exercise (control group). The study will be conducted at Hospital Putrajaya, a main referral center for stroke cases in Putrajaya, the federal administrative capital of Malaysia. Database of post-stroke patients which is maintained by the physiotherapy department of the hospital will be used to recruit participants adequately. The flow of the participants through the trial is as shown in the CONSORT diagram (Fig. 1).

### 2.2 Study participants

Eligible participants will be recruited by the main researcher using the following inclusion criteria:

1. Clinically diagnosed either hemorrhagic or ischemic stroke by a medical or neurology physician.
2. Age 40 to 80 years old (to minimise risk of frailty which is prevalent in those above 80).^[28]^
3. Able to perform basic activities of daily living such as walking and turning, stepping up and down steps with or without a walking aid and hold a glass full of water with the non-affected hand.

Exclusion criteria are:

1. Score below 20 on Montreal Cognitive Assessment (MoCA) which indicates the presence of cognitive impairment
2. Presence of other neurology or medical conditions which limit physical function such as Parkinson's disease, severe musculoskeletal disorders, unstable angina or uncontrolled hypertension.
3. Score 4 or more on a Modified Rankin Scale (4: moderate severe disability; 5: severe disability; 6: death)
4. Attended by a home physiotherapist after discharged from in-patient setting or attending a traditional therapy.
5. Has visual field defects

### 2.3 Participants’ allocation

Selected stroke survivors will be randomized into either game-based circuit exercise (experimental group) or conventional circuit exercise (control group) using a stratified randomization method, with the use of sealed opaque envelopes by an independent researcher. Stratification variables are age, consisting of younger (40–59 years) and older adults (60–80 years), and disability level, namely 1 (no significant disability), 2 (slight disability) and 3 (moderate disability). Using the sealed envelope system, recruited participants will be stratified according to the sequence of age and disability level. Participants are then alternately allocated into each trial group according to their age and disability level categories. In this way, both the game-based circuit exercise and conventional circuit exercise trial group will consist of participants with comparable age and disability levels.

### 2.4 Interventions

The interventions in this study will be conducted for 12 weeks. Game-based circuit exercise group will receive a set of exercise tasks consisting of a) resistance exercises including sit to stand, partial squat, step up down, hip raise and heel raise, b) balance exercises including figure of 8 walking, tandem walking, backward walking, walking with instruction and walking with sudden change direction and c) aerobic exercises including punching jab, hook, cross straight and combination punching maneuver with squat and kicking, which aim to improve muscle strength, postural stability and aerobic endurance.

**Figure 2:**
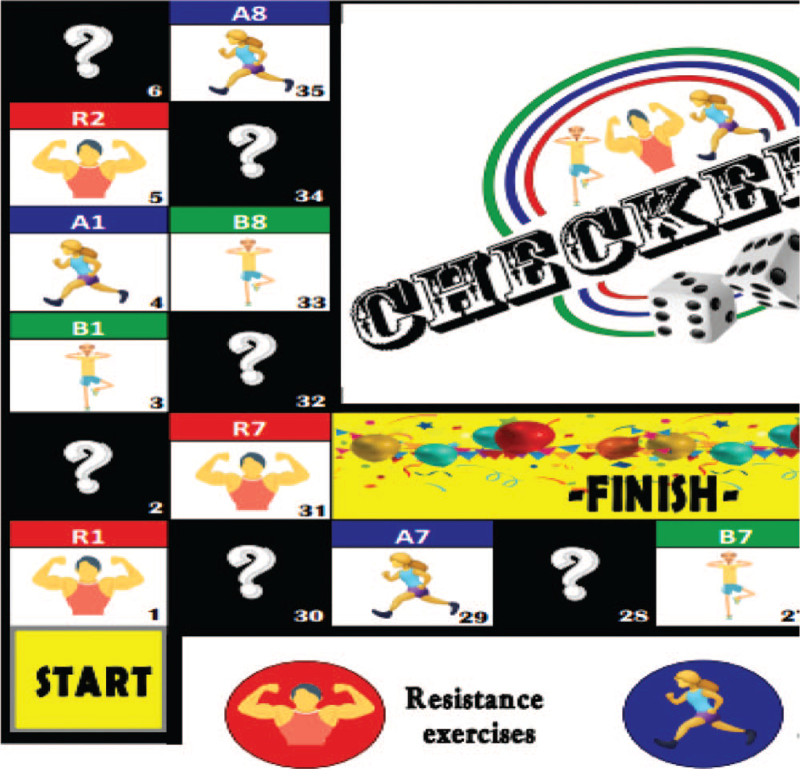
Part of the Checkercise board exercise.

This game-based circuit exercise will use a specially designed exercise board namely Checkercise board (Fig. 2). The design of the Checkercise is similar to the “snake and ladder” game board. To use the Checkercise board, the participants will first be required to place their counter on the space that indicates “start.” Then, they have to take a turn to roll a dice. Next, they have to move their counter forward by several spaces based on the number as shown on the rolled dice. Exercises to be performed by the participants will depend on where their counter land on the board each time the dice is rolled, as each space shows a different exercise task. There is also a possibility of being penalized during the exercise if their counter lands on “penalty spaces,” such as spaces which indicate “slide back a few spaces,” and “move to a certain board number.” The game-based circuit exercises are considered complete when participants arrive at a space that indicates “finish.” The main researcher will supervise the participants in performing the Checkercise board exercise in the group of 4 participants per session. Exercise duration in each space is 2 minutes interspersed by 2 minutes rest with a total of ten exercises to be performed in average for a duration of 40 minutes. Participants will perform the given exercises at a comfortable pace for 2 times per week, under close monitoring by the main researcher. Exercise adherence and the level of exercise intensity (e.g., low, moderate, vigorous) will be monitored using sessions attendance checklist and Borg Scale Rate of Perceived Exertion, respectively. Table 1 shows the frequency, intensity, time and type (FITT) principle of the Checkercise board exercises to be provided to participants in game-based circuit exercise group.

**Table 1:** Description of game-based circuit exercise in the checkercise board.

Formula	Resistance exercise	Balance exercise	Aerobic exercise
	**Repeated sit to stand**	**Walking on balance beam**	**Alternate jab**
Frequency	2 sessions/wk	2 sessions/wk	2 sessions/wk
Intensity	Speed at 50 beats per min	Speed at 30 beats per min	Speed at 100 beats per min
Time	1 min	1 min	1 min
Technique	Alternate seated to standing (without load)	Walking on balance beam (follow rhythm)	Repeated jab punching (follow rhythm)
Progression	Alternate seated to standing (Lifting up 2 kg of dumbbell)	Tandem walking (follow rhythm)	Repeated double jab punching with defense (follow rhythm)
	**Repeated partial squat**	**Figure of 8 walking**	**Alternate hook**
Frequency	2 sessions/wk	2 sessions/wk	2 sessions/wk
Intensity	Speed at 30 beats per min	Speed at 45 beats per min	Speed at 100 beats per min
Time	1 min	1 min	1 min
Technique	Standing, partial squats with arm support as needed (without load)	Figure of 8 walking (follow rhythm)	Repeated hook punching (follow rhythm)
Progression	Standing, partial squats with arm support as needed (Lifting up 2 kg of dumbell/speed at 50 beats per min)	Figure of 8 walking while holding cup of water	Repeated alternate hook with kicking (follow rhythm)
	**Repeated step up & down**	**Walking with instruction**	**Double jab & hook**
Frequency	2 sessions/wk	2 sessions/wk	2 sessions/wk
Intensity	Speed at 70 beats per min	-	Speed at 100 beats per min
Time	1 min	1 min	1 minute
Technique	Standing, alternate steps-ups on the 8-inches step (without load)	Walking & stop (closed eyes in static standing)	Repeated double jab punching with hook (follow rhythm)
Progression	Standing, alternate steps-ups on the 8 inches step board (Lifting up 2 kg of dumbbell/speed at 75 beats per min)	Walking while sudden change instruction	Repeated double jab punching with hook & squat (follow rhythm)
	**Standing; repeated hip raise**	**Walk & touch cones**	**Double jab**
Frequency	2 sessions/wk	2 sessions/wk	2 sessions/wk
Intensity	Speed at 45 beats per min	Speed at 20 beats per min	Speed at 100 beats per min
Time	1 min	1 min	1 min
Technique	Standing, alternate steps-ups on the 8-inches step board (without load)	Walk & touch cones cuboid shape (follow rhythm)	Repeated double jab punching with defense & kick (follow rhythm)
Progression	Standing, alternate steps-ups on the 8-inches step board (Lifting up 2 kg of dumbbell/speed at 50 beats per min)	Walk & touch cones hexagon shape (follow rhythm)	Repeated double jab punching with squat (follow rhythm)
	**Standing; repeated heel raise**	**Backward walking**	**Cross straight**
Frequency	2 sessions/wk	2 sessions/wk	2 sessions/wk
Intensity	Speed at 70 beats per min	Speed at 45 beats per min	Speed at 100 beats per min
Time	1 min	1 min	1 min
Technique	Standing, alternate raises heel (without load)	Backward walking (follow rhythm)	Repeated cross straight punching (follow rhythm)
Progression	Standing, alternate raises heel (Lifting up 2 kg of dumbbell/speed at 75 beats per min)	Backward walking (follow rhythm for 2 min)	Repeated 4 times cross straight punching with squat (follow rhythm)

Meanwhile, participants in the control group will be supervised by 2 physiotherapists to perform the conventional circuit exercises in a group of 5 participants per session. The participants will take turn to perform exercise tasks which are organized in a total of 6 stations, consisted of;

1. cycling,
2. repeated sit to stand,
3. repeated arm curl,
4. repeated hip & heel raise,
5. stepping up and down and
6. obstacle walking.

The exercise duration in each station is 5 minutes interspersed by a 2-minute rest. Similar to the experimental group, all participants in this control group will receive an exercise session of 40 minutes, twice per week for 12 weeks.

### 2.5 Outcomes

Functional performance among the participants will be assessed in term of lower limb strength, dynamic balance and aerobic endurance.

- A 30-second chair rise test will be used to assess lower limb strength. The normal cutoff chair rise repetition is between 14 to 17 and 13 to 15 for healthy male and female aged 55 to 75 years old, respectively.^[29]^ 30-second chair rise test was shown to have high test-retest reliability (ICC = 0.89) and moderate correlation with the leg press test in stroke survivors (*r* = 0.77).^[29]^ Participants will be instructed to repetitively stand up from a 42 cm high and 47.5 cm deep chair while their arms crossed as quickly as possible within 30 seconds. The number of complete sit to stand tasks performed in 30 seconds will be recorded.
- The Dynamic Gait Index (DGI) will be used to evaluate dynamic balance in walking. The normal cutoff mean score is between 23.2 ± 0.9 to 23.9 ± 0.4 for healthy individual aged 50 to 79 years old.^[30]^ The participants will be instructed to perform all 8 walking tasks, which consisted of
  1. walking on level surface,
  2. walking while changing speed,
  3. walking while turning head horizontally,
  4. walking while turning head vertically,
  5. walking with a pivot turn,
  6. stepping over obstacles,
  7. stepping around obstacles, and
  8. stair climbing. Each task is scored using a 4-point ordinal scale ranging from 0 (severe impairment) to 3 (normal).
- The DGI was shown to have high test-retest reliability (ICC = 0.94–0.96)^[31,32]^ and moderate to excellent correlation with the Timed-Up and Go test, 10-meter walk test and Activities-specific Balance Confidence scale in stroke survivors (*r* = 0.68–0.83).^[31]^ The total score ranges from 0 to 24 and a higher total DGI score signifies a higher level of independence in functional mobility among stroke survivors.
- A 6-minute walk test (6 mWT) will be used to measure aerobic endurance. The normative data for mean walked distance is between 357 to 697 meters and 421 to 795 meters for healthy male, and 321 to 621 meters and 392 to 765 meters for female aged 50 to 79 years old.^[33]^ The 6 mWT was shown to have high test-retest reliability (ICC = 0.99)^[34]^ and moderate to excellent correlation with the 2-minute walk test, 12-minute walk test and maximum oxygen uptake in stroke survivors (*r* = 0.66–0.99).^[34,35]^ Participants will be instructed to walk at a comfortable pace along a marked walking course of ten meters in length. The total distance walked in 6 minutes will be recorded in meters.

Motivation level with regard to experimental tasks will be assessed using a Multidimensional Intrinsic Motivation Inventory (IMI). The inventory consists of 4 subscales with a total of 22 questions that can be calculated separately;

1. interest and enjoyment (8 questions);
2. perceived competence (5 questions), perceived choice (5 item) and pressure and tension (5 item).

The IMI has adequate reliability value, indicated by Cronbach's α coefficient (ICC = 0.85).^[36]^ The score ranges from 1 to 7 (1 indicates “not at all true”; 4 indicates “somewhat true”; 7 indicates “very true”) and a higher total score signifies a higher level of motivation level (high 7.00–4.67; average 4.66–2.34; low 2.33–1.00).

Self-efficacy: The stroke self-efficacy questionnaire (SSEQ), a 13-item self-administered questionnaire which designed specifically for stroke survivors will be used to assess the level of self-efficacy among the study participants. SSEQ consists of 2 self-efficacy domains: activity (items 1–8) and self-management (items 9–13).^[37]^ Participants need to rate their confidence level on a 3-point scale, from 0 which indicates “not at all confident” to 3 which represents “very confident.” The total score of SSEQ will be calculated by summation of each item score. Higher score of SSEQ indicates higher self-efficacy level. SSEQ has good reliability, with Cronbach α coefficient value 0.90 and person separation index (PSI) >0.80 for both activity and self-management domains.^[37]^ The SSEQ was shown to have high test-retest reliability in stroke survivors (ICC = 0.86)^[38]^ and excellent correlation with the Fall Efficacy Scale (*r* = 0.803).^[37]^

Quality of life: Short Form-36 (SF-36) will be used to measure quality of life of the participants. SF-36 contains 8 domains (36 questions) and can be calculated separately which is divided into;

1. physical health component represented by physical functioning (10 questions), role limitation due to physical health (4 questions) and pain (2 questions),
2. mental health component represented by social functioning (2 questions), role limitation due to mental problem (3 questions) and emotional wellbeing (5 questions); and
3. physical and mental health component represented by general health (5 questions) and energy/fatigue 4 questions).

The SF-36 was shown to have moderate to high test-retest reliability in stroke survivors (0.57<ICC<0.8)^[39]^ and adequate to good correlation with the EuroQol (*r* = 0.66),^[39]^ EQ-5D Index (*r* = 0.68)^[40]^ and Health Related Quality of Life in Stroke Patient (0.47<r<0.79).^[41]^ The total score ranges from 0 to 100, with higher scores indicating a better quality of life.

### 2.6 Assessment of outcomes

The baseline and therapy outcomes at the end of week 12 of interventions and at a follow up at week 12 and 24 of trial completion will be measured by a therapist who is blinded to the group allocation and trained to conduct the standardized tests. To avoid assessment bias, the recorded baseline assessment data will not be accessible to the blinded assessor during the post-trial and follow-up assessments.

### 2.7 Sample size

The required sample size for this study was estimated using GPower software version 3.0.10. This study will use mixed model analysis of variance (ANOVA) to analyze time, group and interaction effects of the interventions. Therefore, F-test (ANOVA repeated measure, within-between interactions) was chosen and inputted into the system. The study power and alpha value was set at 80% and 0.05, respectively. Based on these inputs, a minimum sample of 82 subjects is required, that is, 41 participants in each trial group.

### 2.8 Data analysis

All data will be entered into IBM Statistical Packages for Social Sciences (SPSS) version 25.0. Intention-to-treat (ITT) analysis approach will be used; all the recruited participants will be included in the outcome analysis. Using ITT approach, missing data will be replaced with “last observation carried forward.” Socio-demography and health profile of the participants will be analyzed descriptively and reported as frequencies (percentages) and mean (standard deviation) or median (inter-quartile range). The effects of the interventions will be analyzed using mixed model ANOVA and reported as main time, group and time-group interaction effects for each outcome variable. Level of significance is set as *P* < .05 for all results.

### 2.9 Ethics and dissemination

This study received ethical approval from the National Medical Ethics and Research Committee (NMERC) (study ID: NMRR–20–2715–57464). NMERC, being an independent research committee under the Malaysia Ministry of Health is also responsible in monitoring the study progress. All participants will be asked to provide an informed consent prior to participating in the study by the main researcher. Participants are allowed to withdraw anytime during the study without providing an explanation. Participants’ personal data will be kept confidential. Investigators in this study will have access to the final trial dataset. The findings of this study will only be published in a peer-reviewed journal.

## 3 Discussions

Circuit exercise training has been shown to benefit stroke survivors in improving their functional performance through exercising in group under therapists’ supervision. However, evidence on including enriched and fun environment into the existing format of circuit exercise to make it more challenging and interesting particularly for stroke survivors is still limited, which warrants more studies on this topic area. The nature of each exercise task in the designed Checkercise board will offer such targeted environment by adding multisensory stimulation and cueing, cognitive stimulation and limb integration training to further increase neuroplasticity potentials.

Further, the presence of fun and enjoyment through competition could be a factor affecting stroke survivors’ motivation and adherence level during exercise training. We believe that the game-based component of our circuit exercise training will induce these positive influences. Within the fun environment, participants will compete and work hard to achieve their best level exercise performance, hence would induce better functional recovery through enhanced neuroplasticity.

This proposed study is expected to fill the gaps in knowledge and strengthen evidence on the benefit of game-based circuit exercise for stroke survivors. This study will also compare game-based circuit exercise with the existing circuit exercise for stroke survivors and provide information whether game-based circuit exercise is a better approach for this group of population in preventing long-term functional problems. It is our hope that findings from this study can be used to enable rehabilitation professionals to design more interesting, creative and effective exercise training in stroke rehabilitation and serve as a reference for future studies regarding game-based circuit exercise.

## Acknowledgment

The authors thank the Research and Ethics Committee of Universiti Kebangsaan Malaysia (UKMMC) and National Medical Research and Ethics Committee, Ministry of Health for the study approval.

## Author contributions

**Conceptualization:** Nor Azlin Mohd Nordin, Mohd Naqiuddin Johar.

**Funding acquisition:** Nor Azlin Mohd Nordin.

**Methodology:** Nor Azlin Mohd Nordin, Mohd Naqiuddin Johar.

**Supervision:** Nor Azlin Mohd Nordin, Aznida Firzah Abdul Aziz.

**Writing – review & editing:** Nor Azlin Mohd Nordin.

**Writing – original draft:** Mohd Naqiuddin Johar.
